# Supplementary material for: Mechanism of Dimer Selectivity and Binding Cooperativity of BRAF Inhibitors
Source: bioRxiv. 2024 Dec 7:2023.12.12.571293. Originally published 2023 Dec 12. Preprint. [Version 3] doi: 10.1101/2023.12.12.571293 (PMC10760002; doi:10.1101/2023.12.12.571293)
Supplement: Supplement 1 [file media-1.pdf]

Mechanism of Dimer Selectivity and Binding Cooperativity of BRAF inhibitors: Supplemental Information

Joseph Clayton[1,2], **Aarion Romany**[1], **Evangelia Matenoglou**[3], **Evripidis Gavathiotis**[3], **Poulikos I. Poulikakos**[4], **Jana Shen**[1]

***For correspondence:**
jana.shen@rx.umaryland.edu (JS)

[1]Department of Pharmaceutical Sciences, University of Maryland School of Pharmacy, Baltimore, MD 21201, United States; [2]Division of Applied Regulatory Science, Office of Clinical Pharmacology, Office of Translational Sciences, Center for Drug Evaluation and Research, United States Food and Drug Administration, Silver Spring, MD 20993, United States; [3]Department of Biochemistry, Department of Medicine, Department of Oncology, Montefiore Einstein Comprehensive Cancer Center, Albert Einstein College of Medicine, New York, NY 10461, United States; [4]Department of Oncological Sciences, Icahn School of Medicine at Mount Sinai, New York, NY 10029, United States

Supplemental tables

Inhibitor	PDB	αC;DFG	BPs	BP H-bonds	αC position	K-E
			**Monomer selective**			
Vemurafenib	5JRQ	CODI	I,II	D594, G596	21.5/21.5	11.5/11.8
Dabrafenib	5CSW	CODI	I,II	K483, D594, G596	21.4/21.8	7.4/8.7
PLX7904	4XV2	CODI	I,II	D594, F595, G596	20.9/20.4	7.5/8.7
			**Equipotent**			
AZ628	4G9R	CIDO	I,II,III	E501, D594	19.5/19.5	3.0/2.6
TAK632	4KSP*	CIDO	I,II,III	E501, D594	18.8/19.0	3.0/3.9
LY3009120	5C9C	CIDO	I,II,III	E501, D594	19.2/19.2	2.7/2.7
Ponatinib	6P3D	CIDO	I,II,III	E501, D594 I573, H574	19.2/19.2	3.0/3.0
Lifirafenib (BGB283)	4R5Y	CIDO	I,II,III	E501	19.0/18.7	5.5/5.1
Tovorafenib (TAK580)	6V34	CIDO	I,II,III	E501, D594	18.6/18.8	2.5/2.9
SB5909885	2FB8*	CIDI	I	E501	18.6/18.3	2.9/3.2
GDC0879	4MNF	CIDI	I	E501	18.4/18.7	2.8/2.9
			**Dimer selective**			
Naporafenib (LXH254)	8F7P*	CIDO	I,II,III	E501, D594	19.3/19.3	3.0/3.0
RAF709	5VAM*	CIDO	I,II,III	E501, D594	19.2/19.1	2.8/2.8
Sorafenib	1UWJ	CIDO	I,II,III	E501, D594	18.8/18.8	2.6/2.6
Belvarafenib	6XFP*	CIDO	I,II,III	E501, D594	19.3/19.3	2.7/2.7
PHI1	6P7G	CIDO	I,II,III	E501, D594, H574	19.1/19.0	2.5/2.5

Table 1. List of the BRAF^{V600E} inhibitors and the structure features of the co-crystal structures in the PDB. The monomer and dimer selectivities of inhibitors in black are based on the experimental data in Ref (***Adamopoulos et al., 2021***) and Ref (***Cotto-Rios et al., 2020***) (PHI1). The monomer and dimer selectivities of inhibitors in red were predicted by us and supported by experiments in Ref (***Karoulia et al., 2016***) (GDC0879) and Ref (***Tkacik et al., 2023***) (Tovorafenib). Note, the PDB entries indicated by an astrisk are co-crystal structures in complex with the wild type BRAF (the BRAF^{V600E} forms are unavailable). All structures contain an inhibitor in each protomer, with the exception of PLX7904; in the PDB entry 4XV1, PLX7904 is only present in protomer A. The αC helix position (in Å) is defined in the main text. Two values refer to the two protomers. The back pockets (BPs) occupied was calculated by KLIFS (***Kooistra et al., 2016***) based on the definition of Liao (***Liao, 2007***). K-E refers to the distance (in Å) between the amine nitrogen of Lys483 and the nearest carboxylate oxygen of Glu501.

System, protomer	αC position (Å)	K-E distance (Å)	DFG dihedral (°)
Apo monomer, A	23.3 ± 1.7	8.1 ± 3.1	214 ± 19
Apo dimer, A	22.2 ± 1.0	6.5 ± 2.9	250 ± 39
Apo dimer, B	22.0 ± 1.7	5.5 ± 2.2	201 ± 24
PHI1 (mixed), apo	21.3 ± 1.0	5.5 ± 3.1	239 ± 41
PHI1 (mixed), holo	18.9 ± 0.7	2.8 ± 0.3	283 ± 24
LY (mixed), apo	22.8 ± 1.7	5.3 ± 2.4	204 ± 35
LY (mixed), holo	19.0 ± 0.5	4.9 ± 1.2	295 ± 13
PHI (holo), A	18.3 ± 0.6	2.8 ± 0.1	285 ± 17
PHI (holo), B	18.7 ± 0.7	2.8 ± 0.1	303 ± 12
LY (holo), A	19.4 ± 0.6	3.4 ± 1.1	301 ± 18
LY (holo), B	19.2 ± 0.5	4.3 ± 1.4	295 ± 13

Table 2. Average value and standard deviation of reported quantities from out dimeric BRAF simulations, separated by system and protomer. Each quantity was calculated for each replica after removing the first 2 μs for equilibration.

Supplemental Figures

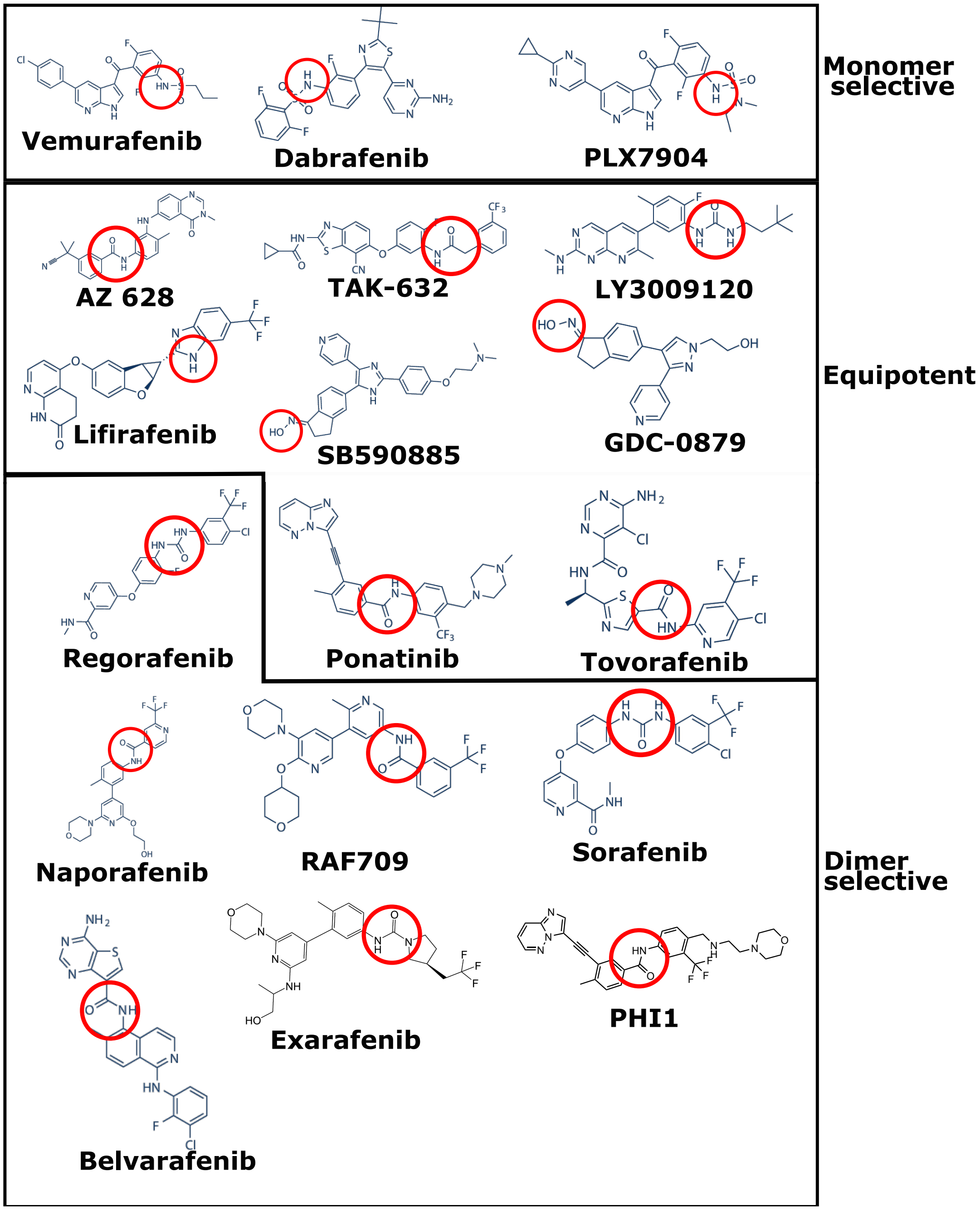

Figure 1. Chemical structures of the monomer-selective, equipotent, and dimer-selective $BRAF^{V600E}$ inhibitors in Supplementary Table 1. The chemical group responsible for forming the hydrogen bonds with E501 and/or D594 are circled in red.

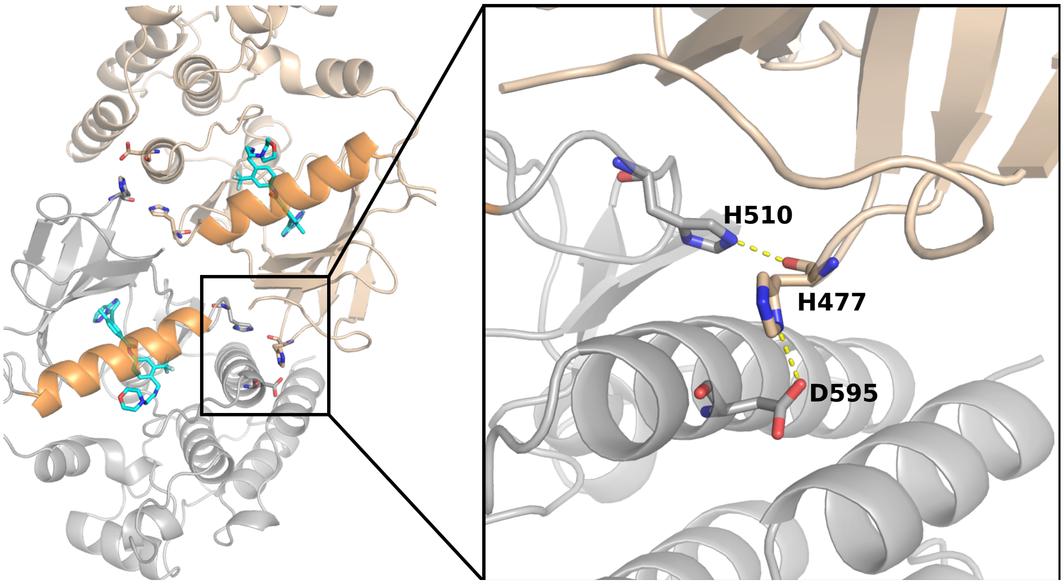

Figure 2. Visualization of the H510-H477-D595 interactions at the dimer interface of BRAF^{V600E}. Left. Visualization of the BRAF^{V600E} dimer (PDB ID: 6P7G) (***Cotto-Rios et al., 2020***). The αC helix is colored brown and PHI1 is colored cyan. The dimer interface residues H477, H510, and D595 are shown in sticks. **Right.** A zoomed-in view of the interactions between H477 in protomer A (light brown color) and H510 or D595 in the protomer B (grey color).

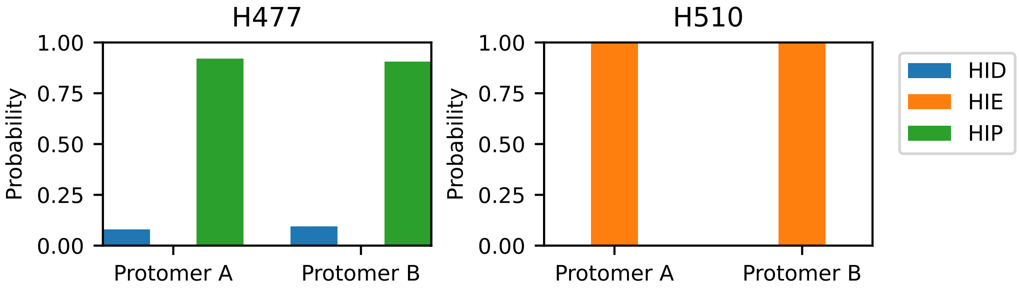

Figure 3. Protonation and tautomer states of the interface histidines determined by the all-atom PME-CpHMD titration. Left. Calculated probabilities of the neutral (HID in blue, HIE in orange) and charged (HIP in green) states for H477 (**left**) and H510 (**right**) at pH 7.5. The probabilities of protonation states for either histidine is nearly identical between the two protomers. As a default in the CpHMD program ***Harris et al.*** (***2022***), the charged HIP state was defined by $\lambda < 0.2$, while the neutral states were defined by $\lambda > 0.8$ and $\chi < 0.2$ (HID) or $\chi < 0.8$ (HIE). The last 5 ns of the replica runs were used for the calculation.

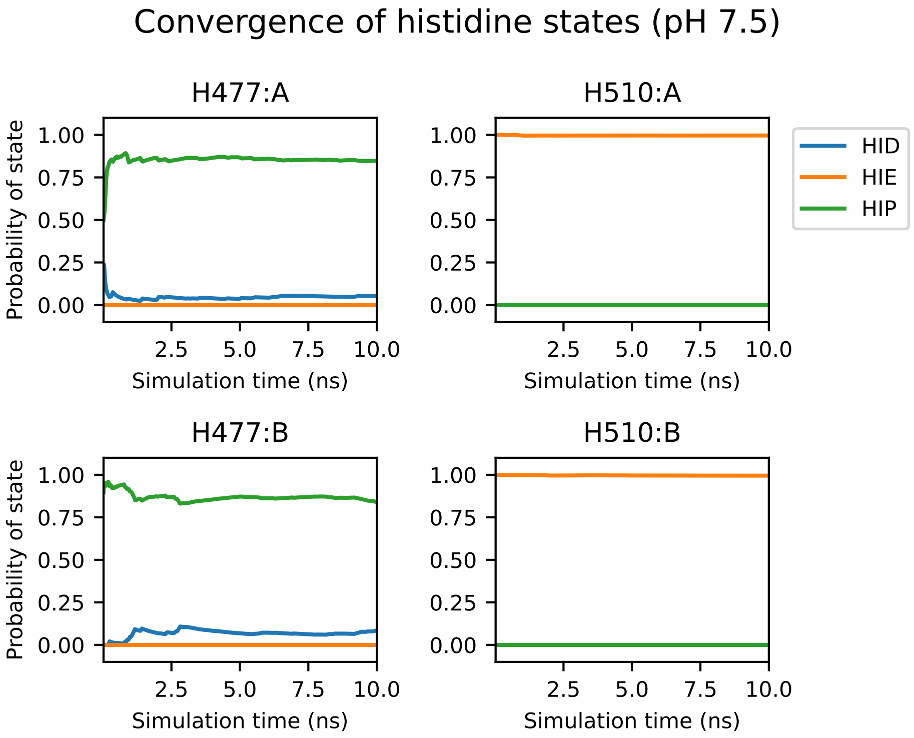

Figure 4. Convergence of the protonation/tautomer states of H477 and H510 at pH 7.5. Running average probability of each protonation/tautomer state for H477 and H510 at pH 7.5 as a function of simulation time (top plots for protomer A and bottom plots for protomer B). Convergence of the probability occurs after roughly 2 ns, with little change after 5 ns.

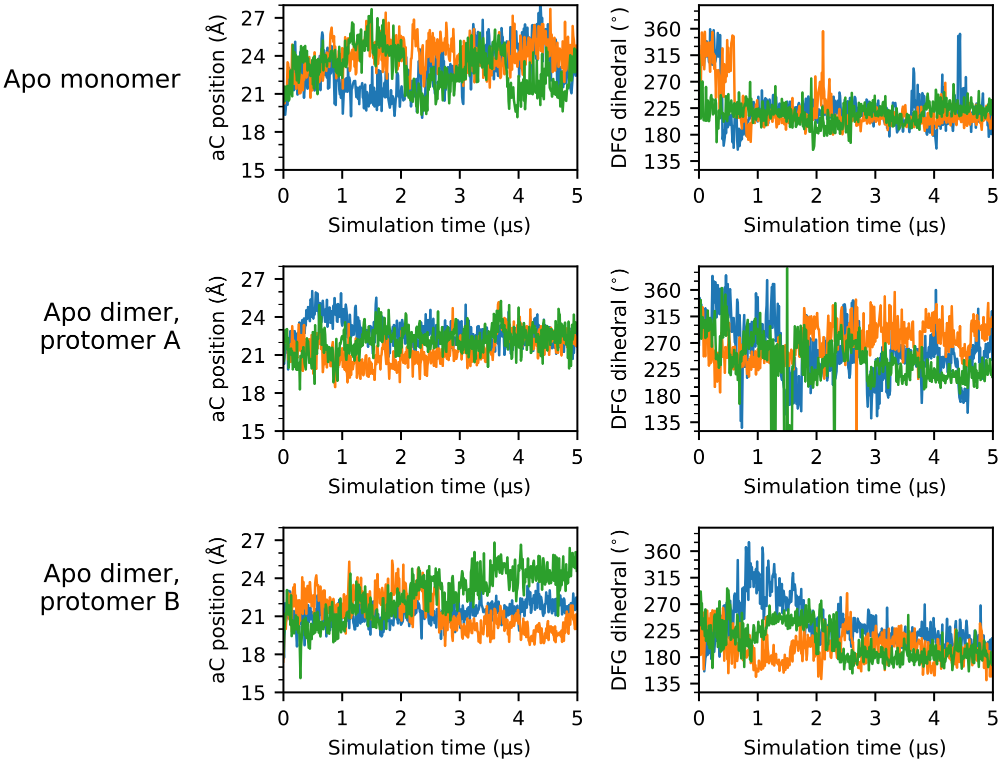

Figure 5. Time series of αC-helix position and DFG dihedral for all apo simulations. Time series plots of the αC-helix position (left column) and DFG dihedral (right column) for each apo protomer simulated. Each of the three replicas are represented as a separate line; for simplicity, a stride of 10 ns was used while plotting.

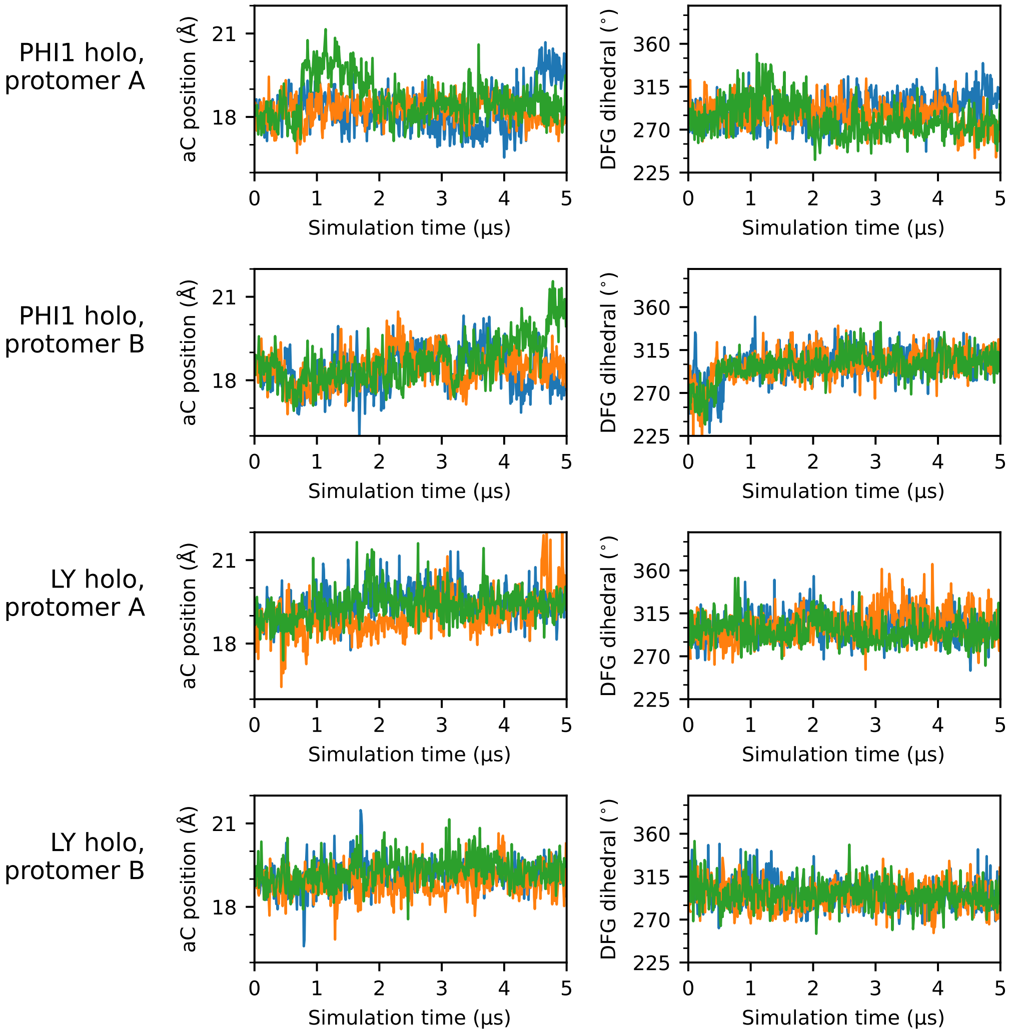

Figure 6. Time series of αC-helix position and DFG dihedral for all holo simulations. Time series plots of the αC-helix position (left column) and DFG dihedral (right column) for each holo protomer simulated. Each of the three replicas are represented as a separate line; for simplicity, a stride of 10 ns was used while plotting.

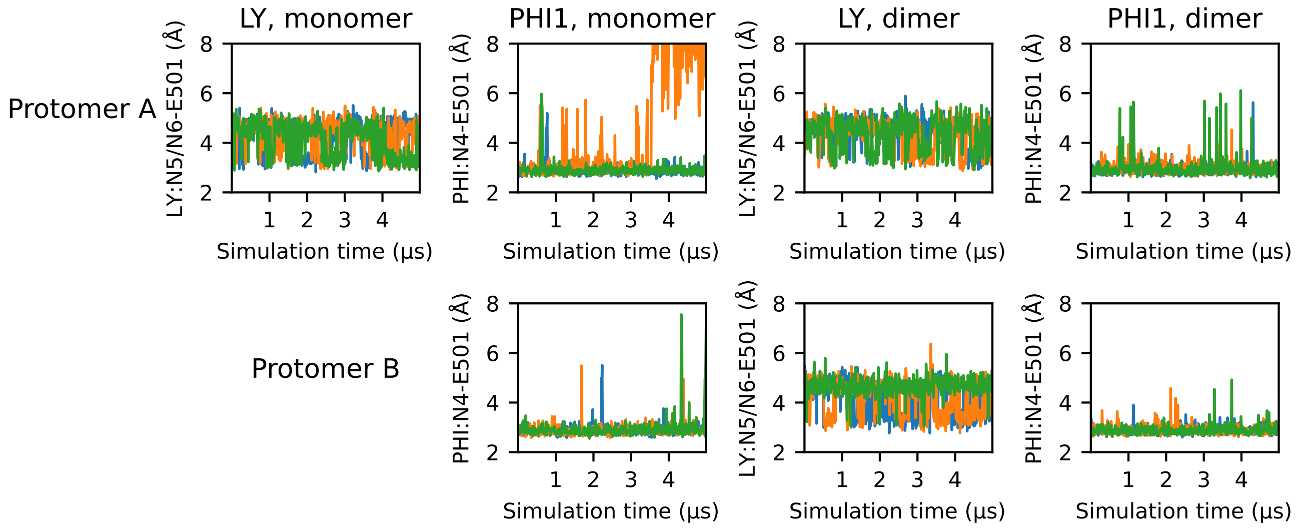

Figure 7. Time series of E501-ligand hydrogen bonding. Time series of the minimum distance between E501 carboxylate oxygens and the proton donor of LY (N5 or N6) and PHI1 (N4). Each of the three replicas are represented as a separate line; for simplicity, a stride of 10 ns was used while plotting.

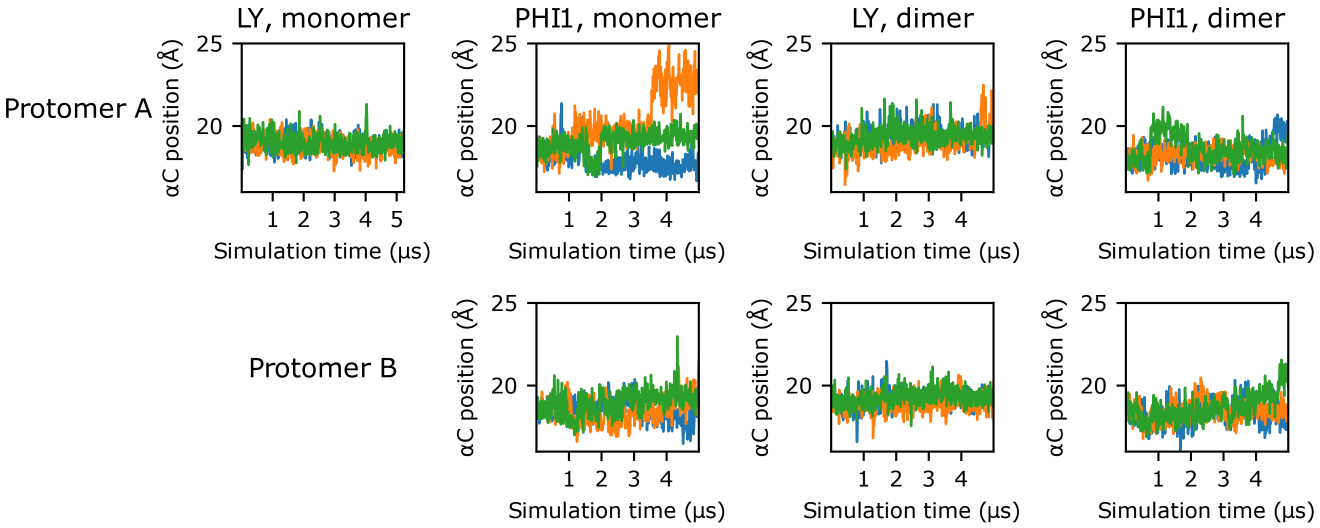

Figure 8. Time series of αC-helix position. Time series of the αC-helix position for inhibited monomer and dimer simulations. Each of the three replicas are represented as a separate line; for simplicity, a stride of 10 ns was used while plotting.

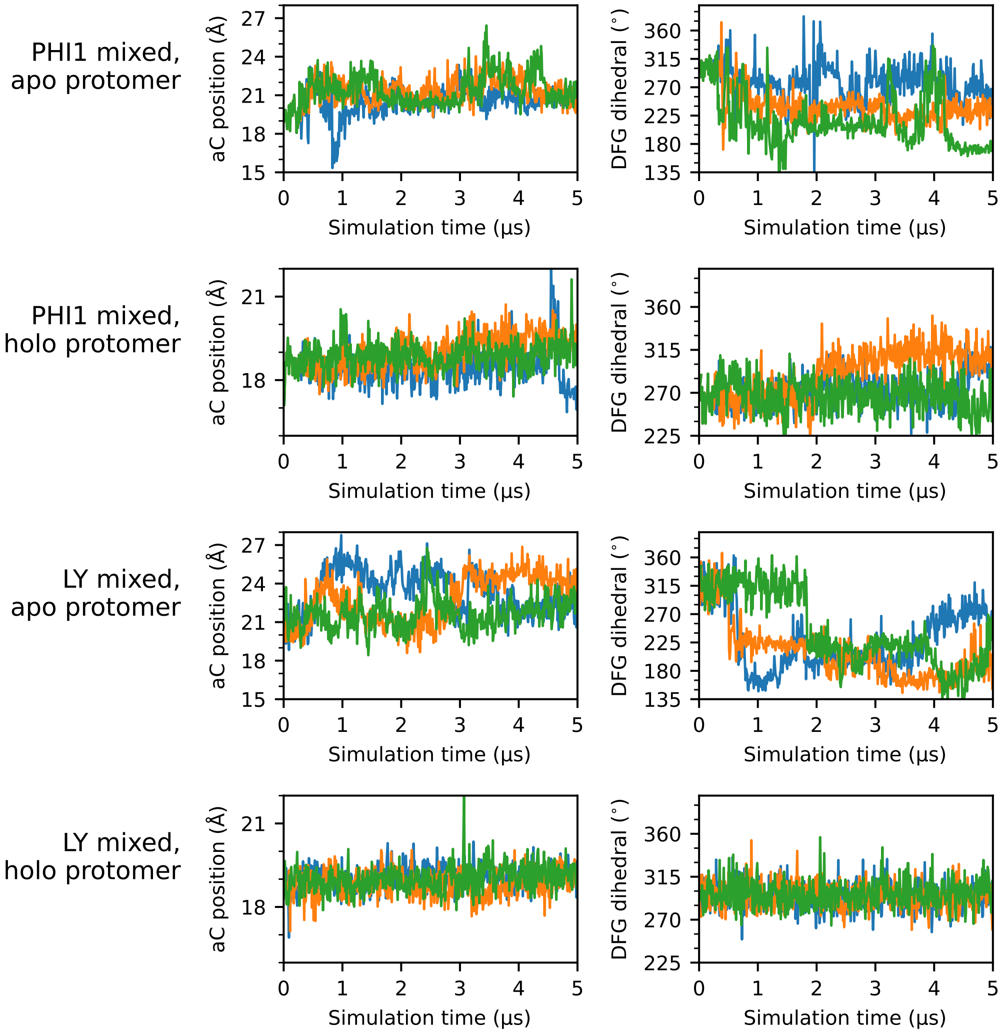

Figure 9. Time series of αC-helix position and DFG dihedral for all mixed simulations. Time series plots of the αC-helix position (left column) and DFG dihedral (right column) for each protomer simulated in the mixed simulations. Each of the three replicas are represented as a separate line; for simplicity, a stride of 10 ns was used while plotting.

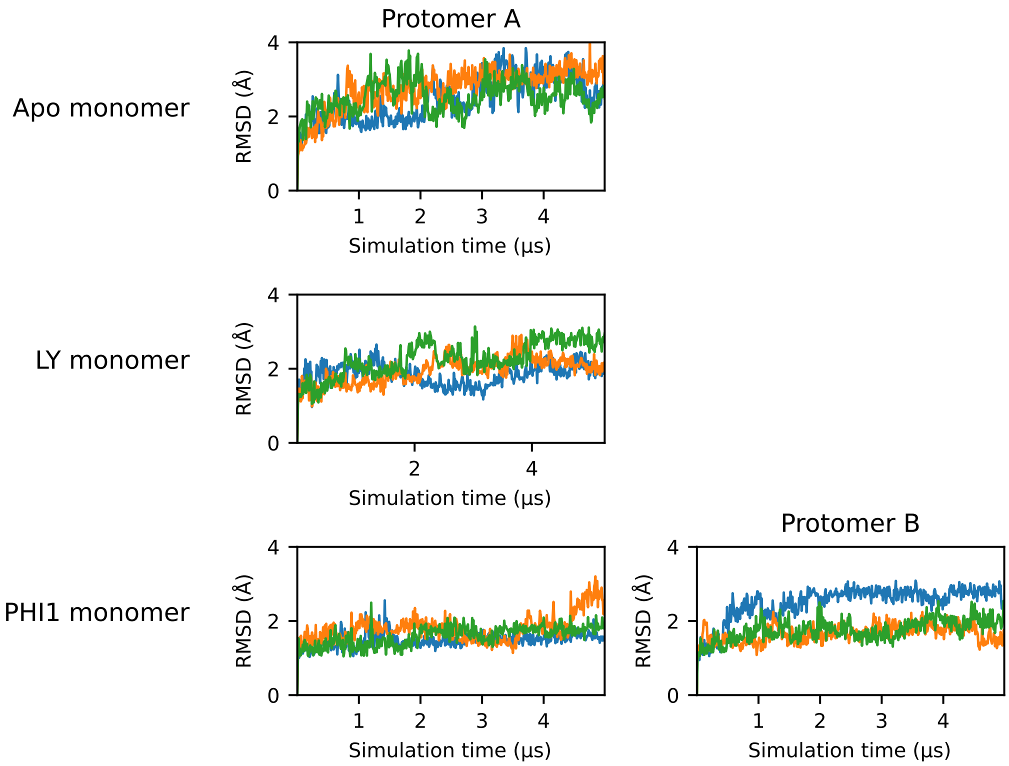

Figure 10. RMSD time series for the monomer simulations. Time series plots of the root-mean-square deviation (RMSD) of the protomer in each monomer simulation. The RMSD was calculated using heavy atoms of the protomer backbone, excluding residues on the a-loop and αC-helix. Each of the three replicas are represented as a separate line; for simplicity, a stride of 10 ns was used while plotting.

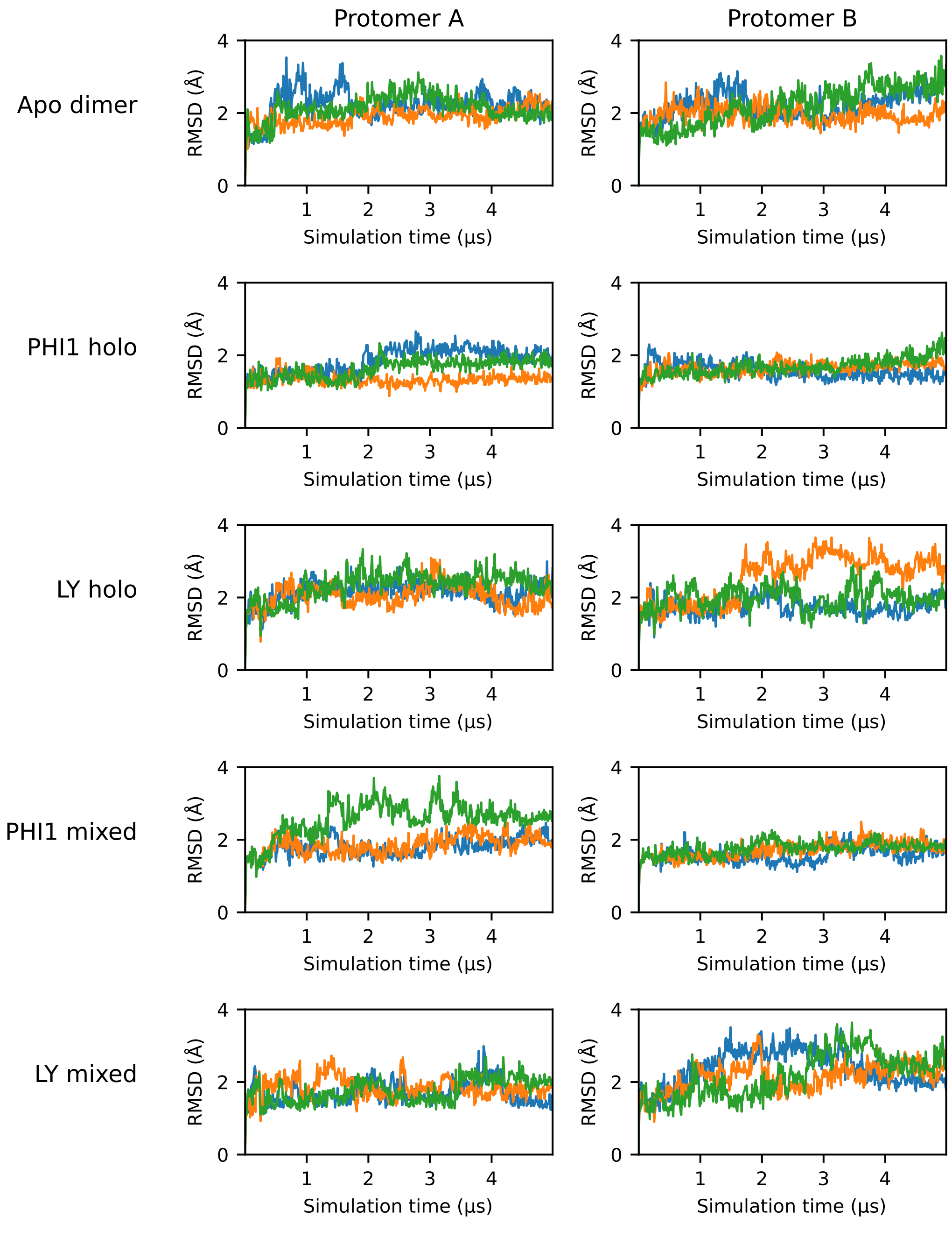

Figure 11. RMSD time series for the dimer simulations. Time series plots of the RMSD of the protomer in each dimer simulation. The RMSD was calculated using heavy atoms of the protomer backbone, excluding residues on the a-loop and αC-helix. Each of the three replicas are represented as a separate line; for simplicity, a stride of 10 ns was used while plotting.

References

Adamopoulos C, Ahmed TA, Tucker MR, Ung PMU, Xiao M, Karoulia Z, Amabile A, Wu X, Aaronson SA, Ang C, Rebecca VW, Brown BD, Schlessinger A, Herlyn M, Wang Q, Shaw DE, Poulikakos PI. Exploiting Allosteric Properties of RAF and MEK Inhibitors to Target Therapy-Resistant Tumors Driven by Oncogenic BRAF Signaling. Cancer Discov. 2021 Jul; 11(7):1716–1735. doi: 10.1158/2159-8290.CD-20-1351.

Cotto-Rios XM, Agianian B, Gitego N, Zacharioudakis E, Giricz O, Wu Y, Zou Y, Verma A, Poulikakos PI, Gavathiotis E. Inhibitors of BRAF Dimers Using an Allosteric Site. Nat Commun. 2020 Dec; 11(1):4370. doi: 10.1038/s41467-020-18123-2.

Harris JA, Liu R, Martins de Oliveira V, Vázquez-Montelongo EA, Henderson JA, Shen J. GPU-Accelerated All-Atom Particle-Mesh Ewald Continuous Constant pH Molecular Dynamics in Amber. J Chem Theory Comput. 2022 Dec; 18(12):7510–7527. doi: 10.1021/acs.jctc.2c00586.

Karoulia Z, Wu Y, Ahmed TA, Xin Q, Bollard J, Krepler C, Wu X, Zhang C, Bollag G, Herlyn M, Fagin JA, Lujambio A, Gavathiotis E, Poulikakos PI. An Integrated Model of RAF Inhibitor Action Predicts Inhibitor Activity against Oncogenic BRAF Signaling. Cancer Cell. 2016 Sep; 30(3):485–498. doi: 10.1016/j.ccell.2016.06.024.

Kooistra AJ, Kanev GK, van Linden OPJ, Leurs R, de Esch IJP, de Graaf C. KLIFS: A Structural Kinase-Ligand Interaction Database. Nucleic Acids Res. 2016 Jan; 44(D1):D365–D371.

Liao JJL. Molecular Recognition of Protein Kinase Binding Pockets for Design of Potent and Selective Kinase Inhibitors. J Med Chem. 2007 Feb; 50(3):409–424. doi: 10.1021/jm0608107.

Tkacik E, Li K, Gonzalez-Del Pino G, Ha BH, Vinals J, Park E, Beyett TS, Eck MJ. Structure and RAF Family Kinase Isoform Selectivity of Type II RAF Inhibitors Tovorafenib and Naporafenib. J Biol Chem. 2023 May; 299(5):104634. doi: 10.1016/j.jbc.2023.104634.
